# Acute Renal Failure and Generalized Weakness in a 75-Year-Old Male With Pauci-Immune Necrotizing Antineutrophil Cytoplasmic Antibody (ANCA)-Associated Vasculitis: A Case Report

**DOI:** 10.7759/cureus.68398

**Published:** 2024-09-01

**Authors:** Wayne A Martini, Lauren B Querin, Nicole R Hodgson, Douglas Rappaport

**Affiliations:** 1 Emergency Medicine, Mayo Clinic, Phoenix, USA

**Keywords:** auto immune, nephrology, emergency medicine, gastric perforation, pauci-immune necrotizing vasculitis, acute kidney injury, anca-associated vasculitis

## Abstract

We describe a 75-year-old male who presented to the emergency department with generalized weakness and was ultimately diagnosed with acute renal failure secondary to pauci-immune necrotizing antineutrophil cytoplasmic antibody (ANCA)-associated vasculitis. The patient’s clinical course was complicated by a perforated gastric ulcer and severe malnutrition, necessitating involvement from multiple specialists. The case highlights the challenges of this rare vasculitis and the complications that can arise from the disease and its treatment.

## Introduction

Antineutrophil cytoplasmic antibody (ANCA)-associated vasculitis (AAV) is a group of rare, systemic autoimmune disorders characterized by inflammation of small to medium-sized blood vessels, often leading to significant organ damage. This condition predominantly affects individuals in the sixth and seventh decades of life and includes several clinical syndromes such as granulomatosis with polyangiitis (GPA), microscopic polyangiitis, eosinophilic granulomatosis with polyangiitis (EGPA), and renal-limited vasculitis. These syndromes are associated with the presence of ANCAs, which target specific proteins within neutrophils, including myeloperoxidase (MPO-ANCA) and proteinase 3 (PR3-ANCA), leading to vascular inflammation [1].

The incidence of AAV varies geographically, with an estimated annual incidence of 10-20 per million globally [2]. In the United States, the incidence of GPA is approximately 13 per million, with a prevalence of 218 per million [3]. AAV is more common in individuals of European ancestry, and the disease often presents earlier in Black patients, who are more likely to have MPO-ANCA. The pathogenesis of AAV is complex, involving genetic susceptibility and environmental triggers such as silica exposure, drug use, and infections [4-6]. Drugs such as levamisole-adulterated cocaine, hydralazine, and propylthiouracil have been implicated in drug-induced ANCA vasculitis [6].

Renal involvement in AAV is particularly concerning, as it often leads to rapidly progressive glomerulonephritis, resulting in acute kidney injury and potential long-term renal dysfunction if not promptly treated [1]. The disease course can be aggressive, stressing the importance of early diagnosis and intervention to improve patient outcomes. However, the management of AAV is complex, requiring a careful balance between controlling the autoimmune process and managing the side effects of immunosuppressive therapies. Understanding the pathophysiology, clinical presentation, and treatment options for AAV is essential for clinicians who find themselves managing patients with this condition.

## Case presentation

A 75-year-old male with no significant past medical history presented to the emergency department (ED) with a five-week history of progressive weakness in his proximal limbs and marked weight loss. He reported increasing difficulty performing daily activities, particularly rising from a seated position. He noted a significant decrease in appetite, resulting in a weight loss of 14 kg over the last two months. The patient had a 40-year history of smoking and occasional marijuana use but denied alcohol or illicit drug use. A recent psychosocial stressor was the death of his son a few weeks before his presentation. 

He reported having been seen at a community clinic for his increased weakness two weeks before his presentation in our ED. At that time, it had been noted that he had some weight loss of about 7 kg and had complete blood count and basic metabolic panel, with the only slight abnormality being a mild increase in his serum creatinine to 1.24 mg/dL (normal range 0.6-1.2 mg/dL). 

On examination, the patient appeared cachectic and exhibited pronounced weakness, particularly in the proximal muscles. Initial laboratory investigations revealed an elevated erythrocyte sedimentation rate of 23 (normal range 0 - 22 mm/1 h), hyponatremia with sodium of 128 mmol/L (normal range 135 - 145 mmol/L), and significantly impaired renal function with a blood urea nitrogen of 106 mg/dL (normal range 8 - 24 mg/dL) and serum creatinine of 5.5 mg/dL. Chest X-ray identified smoking-related emphysematous changes in the lungs and mild aortic dilation. He was admitted to the hospital's Internal Medicine team for his progressive weight loss, weakness, and acute kidney injury.

After admission to the hospital, he was seen by Nephrology, who recommended dialysis and renal biopsy. His screening labs for serum antinuclear antibody returned at 0.8 U (normal range <1.0), c-ANCA returned negative, and proteinase 3 Ab (PR3) returned at <0.2 U (normal range <0.4 U). His serum C3 returned at 137 mg/dL (normal range 75 - 175 mg/dL), and serum C4 returned at 23 (normal range 14 - 40 mg/dL). However, his perinuclear (P-ANCA) labs returned positive on the first day of admission. His MPO serum antibody returned > 8.0 (normal range < 0.4 U). He was started on intravenous (IV) high-dose corticosteroids (500 mg methylprednisolone daily) on hospital day 1. Pathology confirmed pauci-immune necrotizing ANCA-associated large vessel vasculitis without evidence of glomerulonephritis on hospital day 6. 

**Figure 1:**
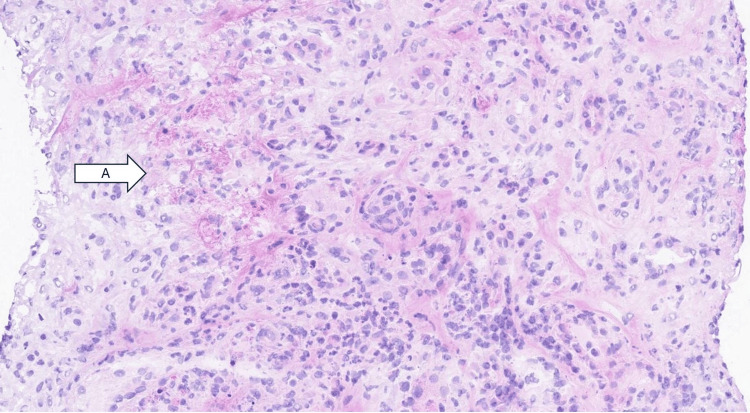
Severe transmural necrosis (arrow A), characterized by extensive destruction of the vessel wall and surrounding tissue necrosis. This finding is a hallmark of pauci-immune/ANCA-associated necrotizing vasculitis, which is further supported by the patient’s positive p-ANCA with MPO specificity. ANCA: Antineutrophil cytoplasmic antibody; MPO: myeloperoxidase

**Figure 2:**
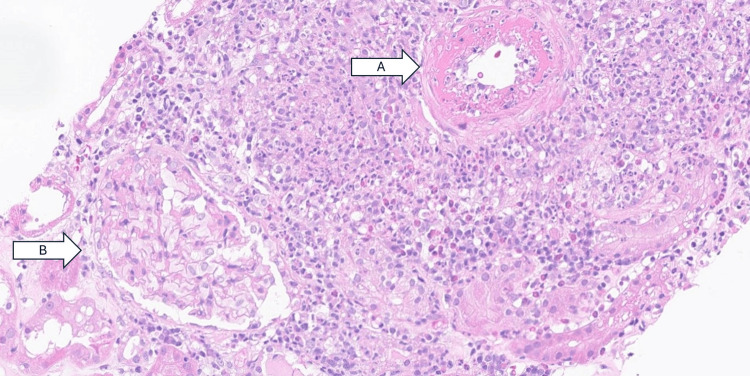
Renal arteriole demonstrating thickening of the wall with narrowing of the lumen, consistent with arteriolosclerosis (arrow A) from longstanding hypertension. Glomerulus (arrow B), which in this biopsy shows no evidence of glomerulonephritis, although the possibility of sampling error cannot be excluded.

The descriptive pathology report from renal biopsy showed that nearly all of the large arteries sampled showed severe transmural necrosis with associated surrounding tissue necrosis (Figure 1). Immunofluorescence was negative for immune complex deposits. Given the patient's positive p-ANCA with MPO specificity, the biopsy supported a diagnosis of pauci-immune/ANCA-associated necrotizing vasculitis. It was very difficult to determine the degree of chronic kidney injury in the background because there was so much interstitial edema, inflammation, and patchy areas of necrosis seen in association with the large vessels. However, overall it appeared to still be mild. No glomerulonephritis was seen; this may be related to sampling error or it may be that this patient's disease was limited to large vessels (Figure 2). Electron microscopy confirmed these findings.

**Figure 3:**
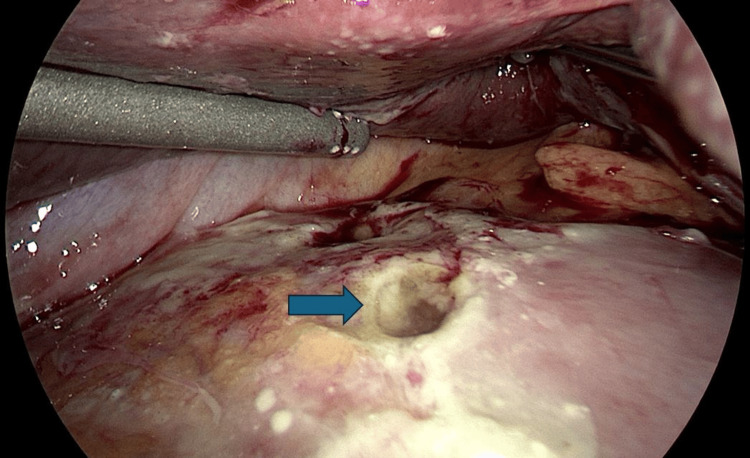
Intraoperative laparoscopic image of a perforated gastric ulcer. The image reveals a clear perforation in the gastric wall, characterized by a distinct, circular defect in the mucosal and muscular layers of the stomach (blue arrow). Surrounding the perforation, there is evidence of fibrinous exudate and inflammation, indicative of acute peritonitis. The presence of necrotic tissue around the ulcer crater suggests ongoing tissue damage. The adjacent gastric mucosa appears erythematous and edematous, further signifying an inflammatory response.

During hospitalization on day 8, the patient developed acute abdominal pain. Subsequent imaging revealed a perforated gastric ulceration (Figure 3), necessitating urgent surgical intervention with diagnostic laparoscopy and gastric perforation repair with a graham patch. Postoperatively, the patient’s recovery was complicated by the need to carefully balance ongoing immunosuppressive therapy with the risk of infection due to recent surgery. He was ultimately discharged to a skilled nursing facility on hospital day 19.

Differential diagnosis 

The differential diagnosis initially included various causes of acute kidney injury, such as prerenal azotemia, exposure to nephrotoxic agents, interstitial nephritis, glomerulonephritis and systemic vasculitis such as granulomatosis with polyangiitis, microscopic polyangiitis, and eosinophilic granulomatosis with polyangiitis. The absence of glomerular involvement on biopsy initially broadened the differential, but the presence of ANCA antibodies and the specific findings on biopsy ultimately confirmed the diagnosis of ANCA-associated vasculitis. The subsequent development of gastric perforation required consideration of the role of vasculitis and corticosteroid therapy in exacerbating the ulceration.

Treatment 

The patient was initially treated with high-dose intravenous corticosteroids to manage the vasculitis, followed by a planned regimen of cyclophosphamide and rituximab. However, this treatment was paused following the diagnosis of a gastric perforation. The patient underwent a modified Graham patch repair for gastric perforation, and postoperatively, immunosuppressive therapy was cautiously reintroduced with avacopan, a selective C5a inhibitor, with close monitoring for potential complications. 

Outcome and follow-up 

The patient’s condition stabilized postoperatively, and his renal function showed partial improvement with liberation from dialysis. Immunosuppressive therapy was cautiously resumed after confirming there was no active infection. During his hospital stay, his serum creatinine levels improved from a peak of 5.59 mg/dL to 3.69 mg/dL (reference range: 0.74 - 1.35 mg/dL) at the time of discharge. He was discharged to a skilled nursing facility. Follow-up appointments with Rheumatology, Nephrology, and General Surgery showed further improvement in his serum creatinine to 3.04 mg/dL. During these visits, he gained two kilograms, had no difficulty with urination, and reported a significant improvement in his appetite.

## Discussion

This case of a 75-year-old male with AAV presents a unique combination of clinical features and complications that underscore the challenges in diagnosing and managing this rare autoimmune disorder. 

Diagnostic challenges 

This case highlights the complexities involved in diagnosing AAV, particularly when renal involvement does not present with the typical glomerulonephritis. The patient presented with progressive weakness, weight loss, and acute kidney injury, leading to a differential diagnosis that included multiple etiologies of failure to thrive which included autoimmune, endocrine, infectious, nutritional, psychosocial, cardiopulmonary, neurologic, oncologic, and renal disorders.

The absence of glomerulonephritis on renal biopsy, despite the presence of pauci-immune necrotizing vasculitis with large vessel involvement, is unusual and complicates the diagnostic process. The presence of high titers of MPO-ANCA ultimately confirmed the diagnosis, yet the lack of glomerular involvement suggests a potentially different pathophysiological mechanism or a sampling error, as seen in similar cases.

Treatment complexities 

Management of AAV is complicated by the need to balance effective immunosuppressive therapy with the risks of adverse effects, such as infection or, in this case, gastrointestinal (GI) perforation. High-dose corticosteroids are standard in the initial treatment phase, often followed by cyclophosphamide or rituximab. However, in this patient, the development of a perforated gastric ulcer while on corticosteroid therapy necessitated a pause in the administration of cyclophosphamide and rituximab. Although it was not given in this case, GI prophylaxis with pantoprazole may help to prevent ulcer formation.

ANCA vasculitis management continues to evolve. Traditionally for patients experiencing organ or life-threatening ANCA vasculitis, 500 mg to 3 g of IV methylprednisolone has been used followed by 1 mg/kg of oral prednisone. Continued research in the last two decades has investigated different approaches to reduce corticosteroid utilization.

The Rituximab versus Cyclophosphamide for ANCA-Associated Vasculitis (RAVE) trial showed that rituximab was not inferior to daily cyclophosphamide for induction of remission in severe ANCA-associated vasculitis and may be superior in relapsing disease, allowing for successful tapering of prednisone by five months [7]. Improved outcomes in the utilization of rituximab compared to cyclophosphamide led to further research for rapid titration of corticosteroids. Among 49 patients who received a combination cyclophosphamide-rituximab infusion, rapid glucocorticoid withdrawal (between one and two weeks) reduced severe adverse events with effective remission induction compared with previous European Vasculitis Society (EUVAS) trials [8]. Combined induction with corticosteroids, rituximab, and low-dose IV cyclophosphamide was studied in a cohort of 66 patients with biopsy specimen-proven renal ANCA vasculitis [9]. Compared with propensity-matched patients in EUVAS trials, 94% of patients achieved remission in six months. Additionally, the combination treatment had lower death rates (HR, 0.29; 95% CI, 0.125 to 0.675), progression to ESRD (HR, 0.20; 95% CI, 0.06 to 0.65), and relapse rates (HR, 0.49; 95% CI, 0.25 to 0.97).

Recent advances in the management of AAV have focused on reducing the cumulative dose of glucocorticoids, as evidenced by the findings of the Plasma Exchange and Glucocorticoids in Severe ANCA-Associated Vasculitis (PEXIVAS) trial, which compared standard-dose or reduced-dose oral glucocorticoid regimens combined with one week of daily plasma exchange in severe ANCA vasculitis [10]. At six months, the reduced-dose group had 60% less glucocorticoid exposure. Further research exists exploring the utilization of avacopan, an orally administered, selective C5a receptor inhibitor, as a substitute to replace oral glucocorticoids without compromising efficacy. Initial trials have shown that in combination with cyclophosphamide and rituximab, C5a receptor inhibition with avacopan was effective in replacing high-dose glucocorticoids in treating vasculitis [11].

## Conclusions

The management of AAV is a complex disease that requires an advanced and continually changing approach that balances the need for aggressive immunosuppressive therapy with the risks of complications. This article reviews the literature regarding the treatment of this rare disease and highlights the importance of close monitoring due to the high potential for the development of adverse reactions including, but not limited to, gastric perforation. Close monitoring and adaptation of the treatment plan, as well as consideration of different strategies that could potentially reduce utilization of high-dose corticosteroids, optimize patient outcomes while reducing complications and steroid dependence. 
